# Increased mortality in hematological malignancy patients with acute respiratory failure from undetermined etiology: a *Groupe de Recherche en Réanimation Respiratoire en Onco*-*Hématologie* (Grrr-OH) study

**DOI:** 10.1186/s13613-016-0202-0

**Published:** 2016-10-25

**Authors:** Adrien Contejean, Virginie Lemiale, Matthieu Resche-Rigon, Djamel Mokart, Frédéric Pène, Achille Kouatchet, Julien Mayaux, François Vincent, Martine Nyunga, Fabrice Bruneel, Antoine Rabbat, Pierre Perez, Anne-Pascale Meert, Dominique Benoit, Rebecca Hamidfar, Michael Darmon, Mercé Jourdain, Anne Renault, Benoît Schlemmer, Elie Azoulay

**Affiliations:** 1 Réanimation médicale, Hôpital Saint Louis, APHP, Paris, France; 2 Informatics and biostatistics, Hôpital Saint Louis, APHP, Paris, France; 3 Réanimation, Institut Paoli Calmettes, Marseille, France; 4 Réanimation médicale, Hôpital Cochin, APHP, Paris, France; 5 Service de Reanimation Médicale et Médecine Hyperbare, CHU Angers, Angers, France; 6 Service de Pneumologie et Réanimation, Groupe Hospitalier Pitié-Salpêtrière, APHP, Paris, France; 7 Réanimation polyvalente, Hôpital Avicenne, APHP, Bobigny, France; 8 Réanimation polyvalente, Hôpital V.Provo, Roubaix, France; 9 Réanimation polyvalente, Hôpital de Versailles, Le Chesnay, France; 10 Réanimation médicale, Hôpital Brabois, Nancy, France; 11 Service des Soins Intensifs Medico-Chirurgicaux et Oncologie Thoracique, Institut Jules Bordet, Brussels, Belgium; 12 ICU, Ghent University Hospital, Ghent, Belgium; 13 Réanimation médicale, CHU de Grenoble, Grenoble, France; 14 Réanimation polyvalente, CHU de Saint-Etienne, Saint-Piest-en-Jarrez, France; 15 Réanimation polyvalente, CHRU de Lille, Lille, France; 16 Réanimation et Urgences Médicales, CHU de la Cavale Blanche, Brest, France; 17 Medical Intensive Care Unit, Hôpital Saint-Louis, ECSTRA Team, Biostatistics and Clinical Epidemiology, UMR 1153 (Center of Epidemiology And Biostatistics Sorbonne Paris Cité, CRESS), INSERM, Université Paris Diderot Sorbonne, 1, Avenue Claude Vellefaux, 75010 Paris, France

**Keywords:** Acute respiratory failure, Hematological malignancies, Outcome, Etiologies, Bronchoalveolar lavage, Diagnostic strategy

## Abstract

**Background:**

Acute respiratory failure (ARF) is the most frequent complication in patients with hematological malignancies and is associated with high morbidity and mortality. ARF etiologies are numerous, and despite extensive diagnostic workflow, some patients remain with undetermined ARF etiology.

**Methods:**

This is a post-hoc study of a prospective multicenter cohort performed on 1011 critically ill hematological patients. Relationship between ARF etiology and hospital mortality was assessed using a multivariable regression model adjusting for confounders.

**Results:**

This study included 604 patients with ARF. All patients underwent noninvasive diagnostic tests, and a bronchoscopy and bronchoalveolar lavage (BAL) was performed in 155 (25.6%). Definite diagnoses were classified into four exclusive etiological categories: pneumonia (44.4%), non-infectious diagnoses (32.6%), opportunistic infection (10.1%) and undetermined (12.9%), with corresponding hospital mortality rates of 40, 35, 55 and 59%, respectively. Overall hospital mortality was 42%. By multivariable analysis, factors associated with hospital mortality were invasive pulmonary aspergillosis (OR 7.57 (95% CI 3.06–21.62); *p* < 0.005), use of invasive mechanical ventilation (OR 1.65 (95% CI 1.07–2.55); *p* = 0.02), a SOFA score >7 (OR 3.32 (95% CI 2.15–5.15); *p* < 0.005) and an undetermined ARF etiology (OR 2.92 (95% CI 1.71–5.07); *p* < 0.005).

**Conclusions:**

In patients with hematological malignancies and ARF, up to 13% remain with undetermined ARF etiology despite comprehensive diagnostic workup. Undetermined ARF etiology is independently associated with hospital mortality. Studies to guide second-line diagnostic strategies are warranted.

*ClinicalTrials.Gov* NCT01172132

**Electronic supplementary material:**

The online version of this article (doi:10.1186/s13613-016-0202-0) contains supplementary material, which is available to authorized users.

## Background

Prevalence of cancer increases steadily over time [1] and is a leading cause of death worldwide, especially in developed countries [2]. This increasing prevalence may be explained by improved sensitivity of diagnostic tests, enhanced efficacy and reduced toxicities of chemotherapy regimens [3], recently released targeted therapies and new drugs contributing to increase overall survival [4], as well as recent advances in stem cell transplantations [5].

Acute respiratory failure (ARF) is the most frequent complication in hematological malignancy (HM) patients, with an incidence reaching 50% [6, 7], leading to high rate of ICU admission [8–11]. Although prognosis of critically ill HM patients improved over the last years [12–17], ARF remains associated with high mortality [8]. Strikingly, mortality rate in that setting is related to ARF etiology [18–20], suggesting that the diagnostic strategy could impact on outcomes.

ARF etiologies are numerous [6, 20–25] mainly related to bacterial or opportunistic infections, underlying disease-specific infiltration [26], or drug-related pulmonary toxicity [27]. Age and associated comorbidities also account for less specific ARF causes such as COPD exacerbation or cardiac pulmonary edema (CPE) [28]. Therefore, diagnosis of ARF could be difficult to identify. Then, a careful, accurate and efficient diagnostic strategy should be implemented to increase diagnostic rate [29, 30]. For instance, a clinical approach easily feasible at the bedside can both assess the most likely diagnosis and guide the best diagnostic approach using noninvasive diagnostic tests or fiberoptic bronchoscopy with bronchoalveolar lavage (BAL) (the DIRECT approach [6, 31]). In that strategy, invasive diagnostic tests, including BAL, would be performed only for specific situations [32–34].

ARF etiology remains undetermined despite a comprehensive diagnostic workup in up to 25% of the cases [33, 35]. Moreover, several studies have reported that immunocompromised patients with undetermined ARF etiology have an increased mortality [18, 32, 36]. However, this finding has never been properly assessed in a large study. Higher mortality for patients with undetermined diagnosis could lead to reinforce our willingness to improve overall diagnostic strategy or to develop new diagnostic tools before discussing lung biopsy.

We sought to appraise the relation between an undetermined ARF etiology and mortality in a large cohort of critically ill hematology patients. The second objective of this study was to assess the yield of invasive procedures, such as BAL, in recent years.

## Patients and methods

 This research is a post hoc analysis of a prospective cohort including 1011 hematological patients admitted to ICU (a study led by the Groupe de Recherche en Réanimation Respiratoire en Onco-Hématologie (GRRR-OH)). As previously published [8], fifteen French and two Belgian ICUs, all familiar with the management of critically ill HM patients, included patients in this cohort within a 16-month period (January 2010–May 2011). The study was approved by the appropriate ethics committees in France (CEERB Bichat, 0235) and Belgium (a different IRB for Brussels and Ghent) and declared on clinical trial (ClinicalTrials.Gov: NCT01172132). All patients or relatives were informed and consented to participate in the study.

Briefly, in the prospective cohort, all patients admitted to ICU with HM were included in the study regardless of the reason of admission. HM was considered if initial diagnosis was performed or relapse occurs within 5 years before ICU admission. Patients’ characteristics at admission and during ICU stay were prospectively collected in a dedicated electronic form. For this post hoc study, only patients with acute respiratory failure (ARF) at ICU admission were analyzed. ARF was defined by an oxygen saturation <90% or PaO2 <60 mmHg on room air, tachypnea (respiratory rate >30/min) or labored breathing, starting within the last 72 h before ICU admission. ARF patients were included regardless of their need for high flow oxygen, noninvasive or invasive mechanical ventilation (IMV).

Patients underwent a global comprehensive assessment to identify ARF etiologies. Invasive or noninvasive diagnostic strategy was performed according to clinical evaluation and CT findings, as previously described [33]. Noninvasive diagnostic tests were blood culture, PCRs for HSV and CMV, serum aspergillus galactomannan, sputa examination, urine antigen tests for *Streptococcus pneumoniae* and *Legionella pneumophila*, nasopharyngeal aspirate or swab including atypical bacteria tests, blood cultures and echocardiography (see Additional file 1). Diagnostic tests were analyzed if they were performed between 2 days before and 4 days after ICU admission. Considering that all participating centers were used to manage such immunocompromised critically ill HM patients and participated in previous studies concerning the diagnostic management of these patients [32, 33], diagnostic strategy performed was the one of the previous studies [33].

Four diagnostic categories were a priori defined by the investigators, based on the need of specific patient’s management (i.e., antibiotics, chemotherapy and steroids, or antifungal, antipneumocystis or antiviral agents) and on previous studies from this group [33]. For each patient, three investigators (EA, VL and FV) analyzed the charts blinded from the diagnosis established by clinicians in charge. Cases of disagreement were then discussed among the three investigators until a consensus could be reached. For 17 patients, no consensus could be reached, the complete charts were analyzed and the diagnosis retained by 2 of the 3 investigators was kept. For 5 patients, the three investigators concluded on a different diagnosis, and no consensus could be reached. These patients were left with an undetermined diagnosis.

Among the four diagnostic categories, the first group included patients with pneumonia as defined by a clinically or microbiologically documented low respiratory tract infection. Clinically documented infection referred to patients with all clinical and radiological criteria for bacterial pneumonia without any microbiological positive results [33]. Microbiologically documented infections were pneumonia with a positive bacterial or viral result from blood cultures (including HSV pneumonia), sputa, nasopharyngeal aspirates, tracheal aspirates or BAL. The second group included patients with non-infectious diagnoses, mostly corresponding to cases of ARF from pulmonary infiltration by the malignancy [26], CPE and drug-related pulmonary toxicity [27]. The third group included patients with ARF from opportunistic infections corresponding to invasive pulmonary aspergillosis according to EORTC criteria [37], proven pneumocystis pneumonia (positive direct examination or immunofluorescence on induced sputa or BAL) and other invasive fungal infections, CMV infections and parasitic infections [33]. Last, patient had undetermined diagnosis when no diagnosis could be made despite noninvasive diagnostic tests, bronchoscopy and BAL when performed and a global patient’s assessment by the ICU team and the hematologist consultant (see Additional file 1).

Results were expressed as median and 25th and 75th quartiles [Q1–Q3] for continuous data and numbers and percentages for categorical data. Marginal association between single variables and outcome was assessed by Wilcoxon rank-sum tests for quantitative variables and Fisher’s exact test or Chi-square test with Yates’ continuity correction for categorical variables when Fisher’s exact test was computationally impossible.

Factors associated with mortality were assessed using multivariate logistic model. Variables associated with the outcome in the first study [8] (poor performance status, Charlson comorbidity index, recipient of allogeneic hematopoietic stem cell transplantation (AHSCT), complete or partial remission, time for hospital to ICU admission <24 h, SOFA score, admission for cardiac arrest, admission for acute respiratory failure, organ infiltration by the malignancy and invasive pulmonary aspergillosis) were included in the model. Invasive mechanical ventilation and undetermined diagnosis were included in the model for this study. A selection procedure was performed using a backward algorithm with a stopping criteria defined by p values below 0.05 for all variables included in the model. Odds ratios of variables present in the final model are given with their 95% confidence intervals. Goodness-of-fit test of the final model was checked using the le Cessie–van Houwelingen test statistic [38].

Survival curves were obtained using the Kaplan–Meier estimator. Differences between survivals were tested using log-rank test. All tests were two sided at the 0.05 significance level. Analyses were performed using R statistical package (http://www.R-project.org).

## Results

**Fig. 1:**
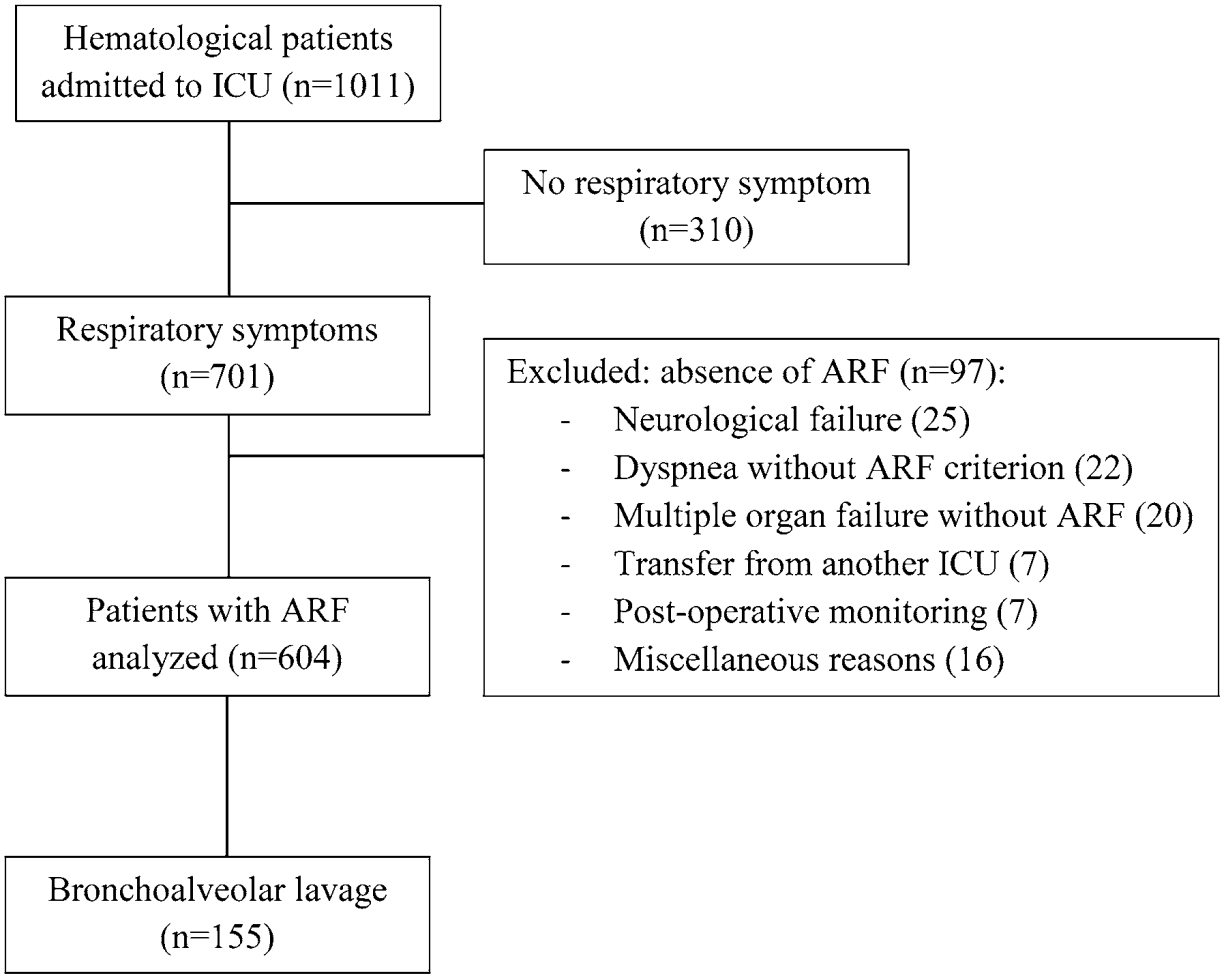
Patients flow diagram. Patients with respiratory symptoms were excluded if they didn’t reach any pre-defined ARF criterion. *ICU* Intensive care unit, *ARF* Acute respiratory failure

Seven hundred and one HM patients with respiratory symptoms were reviewed, including 604 with acute respiratory failure who were included in final analysis (Fig. 1). Our study population included mainly males (*n* = 368, 61%) with a median age of 60 [50–70]. The most prevalent HM patients were acute myeloid leukemia (AML, *n* = 168, 28%) and non-Hodgkin lymphoma (NHL, *n* = 164, 27%). Complete or partial remission concerned 138 (24%) patients, and 107 (17.7%) patients were AHSCT recipients. Most of patients (72%) received antibiotics before ICU admission and/or prophylaxis (15.6%). Altered performance status concerned 124 (20.4%) patients, 182 (30%) patients were neutropenic and 270 (47%) patients had at least 3 organ dysfunctions according to SOFA definition [39]. At ICU admission, the median respiratory rate was 32 [26–38]/min, oxygen flow was 7 [3–15] L/min and two-thirds of the patients had 2 or more quadrants involved on chest X-ray. Noninvasive ventilation was started in 205 (33.9%) patients among whom 65 required secondary intubation. A total of 250 patients (41.4%) needed IMV at day 1. Patient’s characteristics are described in Table 1.

**Table 1:** Study population according to hospital mortality (*N* (%)—median [IQR 25–75])

(*N* (%)—median [IQR 25–75])	Alive at hospital discharge (*n* = 349)	Patients who died (*n* = 255, 42%)	*p* value
Age (year)	60 [50–69]	61 [51–71]	0.43
Malignancy			
Acute myeloid leukemia	90 (25.8%)	78 (30.6%)	0.37
Non-Hodgkin lymphoma	96 (27.5%)	68 (26.7%)	
Myeloma	49 (14%)	32 (12.5%)	
Chronic lymphocytic leukemia	32 (9.2%)	23 (9%)	
Acute lymphoblastic leukemia	19 (5.4%)	19 (7.4%)	
Myelodysplastic syndrome	18 (5.2%)	12 (4.7%)	
Hodgkin’s disease	14 (4%)	4 (1.6%)	
Others	31 (8.9%)	19 (7.5%)	
Disease status at admission			
Earliest phase	120 (34.4%)	88 (34.5%)	0.86
Progression	137 (39.3%)	103 (40.4%)	
Complete or partial remission	83 (23.8%)	55 (21.6%)	
Unknown	9 (2.5%)	9 (3.5%)	
Stem cell transplantation			
Autologous	39 (11.2%)	23 (9.1%)	0.03
Allogeneic	50 (14.3%)	57 (22.4%)	
Poor performance status	55 (15.8%)	69 (27.1%)	0.001
Time from hospital to ICU admission >24 h	159 (46%)	96 (38%)	0.061
Neutropenia	85 (24.4%)	97 (38%)	0.0004
Respiratory rate at admission (/min)	32 [25–37]	35 [25–39]	0.002
Invasive mechanical ventilation at day 1	107 (30.7%)	143 (56.1%)	<0.0001
SOFA score >7	108 (32.2%)	162 (66.7%)	<0.0001
ARF etiologies			
Infectious etiologies	162 (46.4%)	106 (41.6%)	<0.0001
Clinically documented	60 (17.2%)	21 (8.2%)	
Bacterial infection	56 (16%)	54 (21.2%)	
Viral infection	16 (4.6%)	8 (3.2%)	
Other	30 (8.6%)	23 (9%)	
Non-infectious lung involvement	127 (36.4%)	69 (27.1%)	
Malignant infiltrate	25 (7.2%)	18 (7.1%)	
Drug-related lung toxicity	4 (1.1%)	0 (0%)	
Cardiac pulmonary edema	48 (13.8%)	17 (6.7%)	
Other	50 (14.3%)	34 (13.3%)	
Opportunistic infections	28 (8%)	34 (13.3%)	
Invasive pulmonary aspergillosis	6 (1.7%)	24 (9.4%)	
*Pneumocystis jirovecii* infections	18 (5.1%)	4 (1.6%)	
Other invasive fungal infections	3 (0.9%)	2 (0.7%)	
Other	1 (0.3%)	4 (1.6%)	
Undetermined	32 (9.2%)	46 (18%)	

Results were expressed as median and 25th and 75th quartiles [Q1–Q3] for quantitative data and numbers and percentages for categorical data. Marginal association between single variables and outcome was assessed by Wilcoxon rank-sum tests for quantitative variables and Fisher’s exact test or Chi-square test with Yates continuity correction for categorical variables when Fisher’s exact test was computationally impossible

*CR* complete remission, *NA* not available, *PR* partial remission, *IQR* inter-quartile range

Overall, median number of noninvasive diagnostic test performed in each patient was 4 [2–9], including mostly blood cultures (100%), sputum examination (85%), induced sputum examination (14%) and serum aspergillus galactomannan (75%). For 247 (41%) patients, a CT scan was performed. ARF etiologies are reported in Table 1. An infectious etiology was diagnosed in 268 (44.4%) patients, including 110 patients with bacterial pneumonia (18.2%), 81 patients with clinically documented infectious pneumonia (13.4%), 24 patients with viral pneumonia (4%) and 53 patients with miscellaneous etiologies (8.8%). Non-infectious pulmonary involvement was diagnosed in 196 (32.5%) patients, including 65 (10.8%) patients with CPE, 43 patients with lung involvement in underlying disease (7.1%) and 88 patients with miscellaneous non-infectious etiologies (14.6%), among whom 6 patients were diagnosed with intra-alveolar hemorrhage, 1 had hypersensitivity pneumonitis and 4 presented with drug-related pulmonary toxicity. Opportunistic infections were diagnosed in 62 (10.2%) patients, including 30 (5%) patients with invasive pulmonary aspergillosis, 22 (3.6%) patients with pneumocystis pneumonia and 10 (1.6%) patients with other opportunistic diagnoses. For 78 (12.9%) patients, etiology of ARF remained undetermined after the complete diagnosis strategy.

Table 2 reports the comparison between patients with and without undetermined ARF etiology. Patients with undetermined ARF etiology were more frequently AHSCT recipients (26.9 vs 16.4%, *p* = 0.02) and contained a higher proportion of patients with a low performance status (PS > 1, 31 vs 19%, *p* = 0.01). The delay between the diagnosis of the hematological malignancy to ARF and the delay between respiratory symptoms and ICU admission were not different between the 2 groups (487 days [53–1552] vs 216 days [18–1220] (*p* = 0.094) and 32 vs 43.7% of patients were admitted to ICU within the first 24 hours of respiratory symptoms onset (*p* = 0.072), respectively). Also, the proportion of patients with neutropenia and the number of patients who undergone BAL were not different between the two groups (33 vs 29.7%, *p* = 0.60 and 33.3 vs 25.5%, *p* = 0.10, respectively). Patients with an undetermined diagnosis were treated with antibiotics (100%), steroids (65%), antiviral therapy (46%) and antifungal therapy (41%).

**Table 2:** Comparison of patients with and without undetermined ARF etiology (*N* (%)—median [IQR 25–75])

(N (%)—median [IQR 25–75])	Undetermined diagnosis (*n* = 78)	Others (*n* = 526)	*p* value
Age (year)	60.5 [52–72]	60 [50–70]	0.53
Malignancy			
Acute myeloid leukemia	20 (25.7%)	148 (28.1%)	0.26
Non-Hodgkin lymphoma	21 (26.9%)	143 (27.1%)	
Myeloma	18 (23.1%)	63 (12%)	
Chronic lymphocytic leukemia	8 (10.3%)	47 (9%)	
Acute lymphoblastic leukemia	3 (3.8%)	35 (6.6%)	
Myelodysplastic syndrome	4 (5.1%)	26 (5%)	
Hodgkin’s disease	1 (1.3%)	17 (3.2%)	
Others	3 (3.8%)	47 (9%)	
Disease status at admission			
Earliest phase	19 (24.4%)	189 (35.9%)	0.09
Progression	31 (39.7%)	209 (39.7%)	
Complete or partial remission	23 (29.5%)	115 (21.9%)	
Unknown	5 (6.4%)	13 (2.5%)	
Stem cell transplantation			
Autologous	11 (14%)	51 (9.7%)	0.022
Allogeneic	21 (26.9%)	86 (16.3%)	
Performance status 2–4	24 (31%)	100 (19%)	0.025
Delay of admission >24 h	25 (32.1%)	230 (43.7%)	0.072
Neutropenia	26 (33%)	156 (30%)	0.6
Respiratory rate at admission (/min)	35 [28–40]	32 [26–38]	0.13
Invasive mechanical ventilation at day 1	33 (42.3%)	217 (41.3%)	0.88
SOFA score >7	38 (48.7%)	232 (44.1%)	0.39

Results were expressed as median and 25th and 75th quartiles [Q1–Q3] for quantitative data and numbers and percentages for categorical data. Marginal association between single variables and outcome was assessed by Wilcoxon rank-sum tests for quantitative variables and Fisher’s exact test or Chi-square test with Yates continuity correction for categorical variables when Fisher’s exact test was computationally impossible

*CR* complete remission, *NA* not available, *PR* partial remission, *IQR* inter-quartile range

**Fig. 2:**
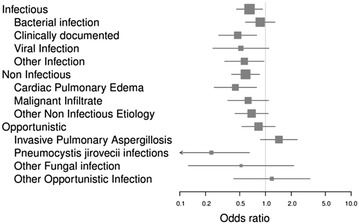
Hospital mortality according to ARF etiology (univariate analysis). Undetermined ARF etiology has been used as a reference

**Fig. 3:**
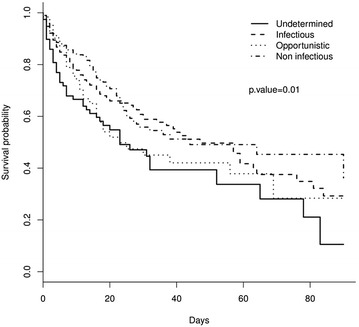
Hospital mortality according to diagnostic category. Survival curves were obtained using the Kaplan Meier estimator. Four diagnostic categories were compared: (1) Infectious: pneumonia as defined by a clinically or microbiologically documented low respiratory tract infection. (2) Noninfectious: patients with non-infectious diagnoses, mostly corresponding to cases of ARF from pulmonary infiltration by the malignancy [26], cardiac pulmonary edema and drug-related pulmonary toxicity [27]. (3) Opportunistic infection: patients with ARF from opportunistic infections (probable or proven invasive pulmonary aspergillosis according to EORTC criteria [37], pneumocystis pneumonia, other cases of invasive fungal infections, CMV infections or parasitic infections [33]. (4) Undetermined diagnosis

Overall ICU mortality and hospital mortality were 30.5 and 42.2%, respectively. Hospital mortality differed significantly across diagnostic categories (univariate analysis, Fig. 2). Namely, mortality ranged from 35% in patients with non-infectious pulmonary involvement to 59% in patients with undetermined ARF etiology and was 40 and 55% for patients with infectious and opportunistic etiologies, respectively. Invasive pulmonary aspergillosis was associated with the higher-case fatality (80%), whereas the lowest mortality was reported in patients with pneumocystis pneumonia (18%) (Figs. 2, 3). End-of-life decision was performed for 152 patients (25%) overall, including 26/78 (33.3%) patients in the undetermined diagnosis group and 126/536 (23.9%) patients in the other groups (*p* = 0.09).

**Fig. 4:**
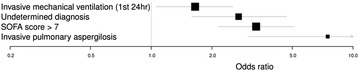
Multivariable analysis of risk factors for hospital mortality. *Box* size is proportional to the accuracy of the estimate. A selection procedure was performed using a backward algorithm with a stopping criteria defined by *p* values below 0.05 for all variables included in the model. Goodness-of-fit test of the final model was checked using the le Cessie–van Houwelingen test statistic

By multivariable analysis (Fig. 4), factors associated with hospital mortality were invasive pulmonary aspergillosis (OR 7.49 (95% CI 3.03–21.37); *p* < 0.005), IMV in the first 24 h of ICU admission (OR 1.65 (95% CI 1.06–2.54); *p* = 0.02), a SOFA score >7 (OR 3.31 (95% CI 2.15–5.13); *p* < 0.005) and an undetermined ARF etiology (OR 2.71 (95% CI 1.59–4.68); *p* < 0.005).

As given in Additional file 1: Table S1, 155 (26%), patients underwent bronchoscopy and BAL. As compared with the no-BAL population, these patients were significantly younger (58 [49–67.5] vs 61 [52–71] year old, *p* = 0.019), with a controlled underlying disease (CR or PR in 31 vs 20% of the patients, *p* = 0.026), more often AHSCT recipients (24.7 vs 15.4%, *p* = 0.004) and mostly non-neutropenic (23.2 vs 32.5% of the patients, *p* = 0.038). At ICU admission, patients who underwent BAL had a more severe respiratory disease (higher respiratory rate (34 [28–40]/min vs 32 [26–38]/min *p* = 0.03), diffuse pulmonary involvement on chest X-ray (involvement of more than 1 quadrant in 71.6 vs 55% of the patients, respectively, *p* = 0.001) and were more often invasively mechanically ventilated in the first 24 h (58.1 vs 35.6%, respectively, *p* < 0.0001). No diagnosis could be performed in 26/155 (16.8%) patients who underwent BAL and in 52/449 (11.6%) of the remaining patients (*p* = 0.13). Hospital mortality rate was 48% for patients who undergone BAL and 40% for those who did not (*p* = 0.13). Strikingly, diagnosis was brought by BAL in 37 (23.9%) patients of the BAL group, among whom BAL was the only positive diagnostic test for 28 (18%) patients (including 9 pneumocystis pneumonias, 9 bacterial pneumonias, 5 viral pneumonias, 2 invasive pulmonary aspergillosis, 1 intra-alveolar hemorrhage, 1 hypersensitivity pneumonitis and 1 pulmonary toxoplasmosis). For 92 patients (59.4%), noninvasive tests were the unique positive investigations. Diagnosis was brought by both techniques in 9 cases (5.8%). More specifically, among the 30 patients with invasive pulmonary aspergillosis overall, 14 diagnoses were performed in the BAL group, in whom 2 patients were diagnosed with BAL, 11 patients were diagnosed with noninvasive techniques and 1 patient was diagnosed with both procedures. Among the 22 pneumocystis pneumonia overall, 13 diagnoses were performed in the BAL group, in whom 9 patients were diagnosed with BAL as sole positive investigation, 2 patients were diagnosed with noninvasive microbiological investigations and 2 patients were diagnosed with both techniques.

## Discussion

In this multicenter study conducted on a prospective cohort of 604 patients with ARF and HM, an undetermined etiological diagnosis was strongly associated with higher hospital mortality. Although previously suggested, this is the largest study that specifically addressed this major clinical question. Moreover, in this study, BAL remained an important diagnostic tool for pneumocystis pneumonia but did not improve diagnostic rate or outcome of ARF.

One of the striking results of the present work is the lower rate of undetermined diagnosis than previously reported (12.9 vs 20–30%) [7, 9, 18, 33, 35]. This result might be related to the recent advances in the management of patients with HM as well as non-immunocompromised patients and the improvement of noninvasive tests [40–45] leading to higher number of diagnoses. By multivariate analysis adjusted on confounders, having an undetermined ARF etiology was independently associated with mortality.

The impact of undetermined ARF etiology on outcome has been assessed only in a single-center study on cancer patients [18]. In that study, patients in whom no ARF etiology could be identified had a 66% mortality rates, and those with known ARF etiology had a 43% mortality rate (*p* = 0.008), in the same ranges than both groups in the present study [18]. Furthermore, a more recent study from our group on patients with solid tumors and HM reported the same finding [32].

Besides undetermined ARF etiology, this study identifies three factors significantly associated with hospital mortality, namely IMV in the first 24 h, invasive pulmonary aspergillosis and a SOFA score >7. IMV is a well-known risk factor for death among hematology patients with ARF [18, 32, 46, 47]. Invasive fungal infections such as pulmonary aspergillosis have also been associated with high-case fatality rates in this population [18, 48].

Noteworthy, diagnostic impact of bronchoscopy and BAL was limited in this study, in agreement with earlier findings [32], even if it was performed in a subgroup of patients. As noninvasive diagnostic tests have been widely used, BAL was the only yielding test in few patients. In a randomized controlled trial published in 2010, we also reported that BAL was diagnostic in only 18% of the patients and that a strategy without bronchoscopy and BAL was not inferior to routine use of BAL [33]. Such low diagnostic yield from BAL may pertain to the number of patients receiving prophylaxis or empirical therapy, to the number of AHSCT recipients in whom all diagnostic tests are less efficient and to the experience of this study group used to manage hematology patients using noninvasive diagnostic tests. However, it should be noted that BAL had high diagnostic yield in pneumocystis pneumonia and remains the only reliable diagnostic tool in patients with alveolar hemorrhage, hypersensitivity pneumonitis, drug-related pulmonary toxicity or acute interstitial pneumonia. Diagnostic contribution of alveolar cellular patterns and cytology still needs additional investigations [49].

This study has several limitations. First, as in a cohort study with no protocolized intervention, not all diagnostic tests could be performed in each patient. Identifying ARF etiology may be related to the number of tests performed. Moreover, patients who died in the first days of ICU admission would not have number of investigation tests. Yet, in this study, patients with undetermined diagnosis died earlier than the others (Fig. 3). Then, undetermined diagnosis might be actually some of undiagnosed infections. Moreover, although all the participating ICUs were high-volume centers used to manage critically ill hematological patients, we could not be sure that diagnosis strategy was the same in all centers. However, rate of undetermined diagnosis was lower than the rate in previous study [33], and we did not find any center effect. Second, BAL was performed according to physician’s decision, and most of patients were intubated before BAL. However, diagnostic yield of bronchoscopy and BAL is in the same ranges than previous reports. Third, these results were obtained in high-volume centers and may not be generalizable to all centers. However, most of hematology patients are managed in highly specialized comprehensive cancer centers with unique collaboration between hematologists, intensivists and other specialists. Fourth, this cohort included a wide variability of patients, in terms of HM, neutropenia, stage of disease, cause of ARF and stem cell transplantation. Last, none of the patients without documented ARF etiology underwent pulmonary biopsy. However, active malignancies and thrombocytopenia, severe hypoxemic pulmonary involvement, associated organ dysfunction and hemostatic disorders precluded this invasive investigation. Nevertheless, mortality related to undetermined diagnosis in this study suggests to reappraise the risk–benefit ratio for these high-risk patients.

## Conclusions

Despite comprehensive diagnostic workout, failure to document ARF etiology occurs in 13% of critically ill hematology patients and is associated with increased mortality. Studies are needed to guide second-line diagnostic strategy and the place of pulmonary biopsy in hematology patients with ARF.

## Additional file

**Additional file 1: Table S1.** Comparison between BAL and No BAL groups *N* (%)—median [IQR 25–75]. Results were expressed as median and 25th and 75th quartiles [Q1–Q3] for quantitative data and numbers and percentages for categorical data. Marginal association between single variables and outcome was assessed by Wilcoxon rank-sum tests for quantitative variables and Fisher’s exact test or Chi-square test with Yates continuity correction for categorical variables when Fisher’s exact test was computationally impossible. CR: Complete remission; NA: Not available; PR: Partial remission; PS: Performance status; IQR: Inter-quartile range.

## Abbreviations

AHSCT: allogeneic hematopoietic stem cell transplantation
ALL: acute lymphoblastic leukemia
AML: acute myeloid leukemia
ARDS: acute respiratory distress syndrome
ARF: acute respiratory failure
BAL: bronchoalveolar lavage
CPE: cardiac pulmonary edema
CR: complete remission
HM: hematological malignancy
ICU: intensive care unit
IMV: invasive mechanical ventilation
NA: not available
NHL: non-Hodgkin lymphoma
PR: partial remission
