# Supplementary material for: Increased mortality in hematological malignancy patients with acute respiratory failure from undetermined etiology: a Groupe de Recherche en Réanimation Respiratoire en Onco-Hématologie (Grrr-OH) study
Source: Ann Intensive Care. 2016 Oct 25;6:102. doi: 10.1186/s13613-016-0202-0 (PMC5080277; doi:10.1186/s13613-016-0202-0)
Supplement: Supplementary file 1 — Additional file 1: Table S1. Comparison between BAL and No BAL groups N (%)—median [IQR 25–75]. Results were expressed as median and 25th and 75th quartiles [Q1–Q3] for quantitative data and numbers and percentages for categorical data. Marginal association between single variables and outcome was assessed by Wilcoxon rank-sum tests for quantitative variables and Fisher’s exact test or Chi-square test with Yates continuity correction for categorical variables when Fisher’s exact test was computationally impossible. CR: Complete remission; NA: Not available; PR: Partial remission; PS: Performance status; IQR: Inter-quartile range. [file 13613_2016_202_MOESM1_ESM.docx]

**Increased Mortality in Hematological Malignancy Patients with Acute Respiratory Failure from Undetermined Etiology. A *Groupe de Recherche Respiratoire en Réanimation Onco-Hématologique* (Grrr-OH) study**

Adrien Contejean MD , Virginie Lemiale MD , Matthieu Resche-Rigon MD, Djamel Mokart MD, Frédéric Pène MD, PhD, Achille Kouatchet MD, Julien Mayaux MD, François Vincent MD, Martine Nyunga MD, Fabrice Bruneel MD, Antoine Rabbat MD, Pierre Perez MD, Anne-Pascale Meert MD, Dominique Benoit MD, PhD, Rebecca Hamidfar MD, Michael Darmon MD, PhD, Mercé Jourdain MD, PhD, Anne Renault MD, Benoît Schlemmer MD PhD, Elie Azoulay MD, PhD.

Supplementary material

**Supplementary method : Criteria for diagnosis of acute respiratory failure in 4 categories**

1/ Pneumonia

Clinically documented pneumonia: This diagnosis refer to patients with all clinical, radiological and outcome criteria for bacterial pneumonia without any microbiological positive results.

Microbiologically documented pneumonia: patients with all clinical and radiological criteria with at least one positive microbiologic specimen:

- Sputum: bacteria >10^6^ CFU/ml and cellularity < 10 epithelial cells/field with > 25 neutrophils/field.
- BAL : bacteria > 10^4^ CFU/ml or positive PCR test for HSV, or for respiratory virus (rhinovirus, VRS, virus influenza, enterovirus, adenovirus, metapneumovirus), or PCR for *Mycoplasma pneumonia*, *Legionella pneumophila*, *Chlamydia pneumonia*)
- Distal protected aspirate : bacteria at a concentration > 10^3^ CFU/ml
- Nasopharyngeal swab: positive result for a respiratory virus with clinical features consistent with viral pneumonia [REF CHEMALY 18] or for *Mycoplasma pneumonia*, *Legionella pneumophila*, *Chlamydia pneumonia*.
- Positive urinary antigen for *Legionella pneumophila*
- Positive urinary antigen for *Streptococcus pneumonia* with criteria for acute pneumonia.

2/ Non infectious

- Cardiac pulmonary edema (CPE): a transthoracic echocardiography was performed at admission. CPE was diagnosed using previously reported criteria[1]
- Alveolar hemorrhage was considered using previously reported criteria[2] among patients with no infection or heart failure.
- Pulmonary infiltration by the malignancy was considered using previously reported criteria[3]

3/ Opportunistic

- Pneumocystis pneumonia: the retrieval of *Pneumocystis jiroveci* in BAL fluid, sputum or induced sputum was considered as confirming the diagnosis. A positive PCR test for *Pneumocystis jiroveci* was not enough to confirm diagnosis.
- Tuberculosis: the retrieval of *Mycobacterium tuberculosis* in BAL fluid, sputum or induced sputum was considered as confirming the diagnosis.
- Invasive pulmonary aspergillosis was diagnosed according to the EORTC criteria[4]
- CMV pneumonia: CMV pneumonia was diagnosed using previously published criteria[5]

**Bibliography**

1. Azoulay É, Mokart D, Lambert J, Lemiale V, Rabbat A, Kouatchet A, et al. Diagnostic strategy for hematology and oncology patients with acute respiratory failure: randomized controlled trial. Am. J. Respir. Crit. Care Med. 2010;182:1038–46.

2. De Lassence A, Fleury-Feith J, Escudier E, Beaune J, Bernaudin JF, Cordonnier C. Alveolar hemorrhage. Diagnostic criteria and results in 194 immunocompromised hosts. Am. J. Respir. Crit. Care Med. 1995;151:157–63.

3. Azoulay E, Fieux F, Moreau D, Thiery G, Rousselot P, Parrot A, et al. Acute monocytic leukemia presenting as acute respiratory failure. Am. J. Respir. Crit. Care Med. 2003;167:1329–33.

4. De Pauw B, Walsh TJ, Donnelly JP, Stevens DA, Edwards JE, Calandra T, et al. Revised definitions of invasive fungal disease from the European Organization for Research and Treatment of Cancer/Invasive Fungal Infections Cooperative Group and the National Institute of Allergy and Infectious Diseases Mycoses Study Group (EORTC/MSG) Consensus Group. Clin. Infect. Dis. Off. Publ. Infect. Dis. Soc. Am. 2008;46:1813–21.

5. Coisel Y, Bousbia S, Forel J-M, Hraiech S, Lascola B, Roch A, et al. Cytomegalovirus and herpes simplex virus effect on the prognosis of mechanically ventilated patients suspected to have ventilator-associated pneumonia. PloS One. 2012;7:e51340.

| **Table S1: Comparison between BAL and No BAL groups N (%) – Median [IQR 25-75]** | | | |
| --- | --- | --- | --- |
|  | **No BAL group** | **BAL group** | **p value** |
| Total | 449 (74%) | 155 (26%) |  |
| Age | 61 [52-71] | 58 [49-67.5] | 0.019 |
| Hemopathy :   - Acute myeloid leukemia - Non Hodgkin lymphoma - Myeloma - Chronic lymphocytic leukemia - Acute lymphoblastic leukemia - Myelodysplastic syndrome - Hodgkin’s desease - Others | 132 (29.4%)  110 (24.5%)  63 (14%)  38 (8.5%)  29 (6.5%)  25 (5.6%)  13 (2.9%)  39 (8.7%) | 36 (23.2%)  54 (34.8%)  18 (11.6%)  17 (11%)  9 (5.8%)  5 (3.2%)  5 (3.2%)  11 (7%) | 0.21 |
| Inaugural pathology  Progression  CR / PR  Unknown | 159 (35.4%)  186 (41.4%)  90 (20%)  14 (3.2%) | 49 (31.6%)  54 (34.8%)  48 (31%)  4 (2.6%) | 0.026 |
| Stem cell transplantation   - Autologous - Allogeneic | 41 (9.1%)  69 (15.4%) | 21 (13.6%)  38 (24.7%) | 0.004 |
| PS 2 - 4 | 91 (20.3%) | 33 (21.3) | 0.88 |
| Delay of admission > 24h | 197 (44%) | 58 (37.9%) | 0.21 |
| Neutropenia | 146 (32.5%) | 36 (23.2%) | 0.038 |
| Respiratory rate at admission (/min) | 32 [26-38] | 34 [28-40] | 0.03 |
| Chest radiography quadrants  0  1  2-4  NA | 77 (19.5%)  70 (17.8%)  247 (55%)  55 | 14 (10%)  15 (10.7%)  111 (71.6%)  15 | 0.001 |
| Invasive mechanical ventilation at day 1 | 160 (35.6%) | 90 (58%) | <0.0001 |
| SOFA score > 7 | 192 (44.6%) | 78 (53%) | 0.23 |
| ARF etiologies  Infectious etiologies   - Clinically documented - Bacterial infection - Viral infection - Other   Non-infectious lung involvement   - Malignant infiltrate - Cardiac pulmonary edema - Drug-related lung toxicity - Other   Opportunistic infections   - Invasive pulmonary aspergillosis - Pneumocystis jirovecii infections - Other invasive fungal infections - Other   Undetermined | 205 (45.7%)  55 (12.3%)  85 (18.9%)  16 (3.6%)  49 (10.9%)  162 (36.1%)  34 (7.6%)  57 (12.7%)  3 (0.7%)  68 (15.1%)  30 (6.6%)  16 (3.5%)  9 (2%)  3 (0.7%)  2 (0.4%)  52 (11.6%) | 63 (40.6%)  26 (16.8%)  25 (16.1%)  8 (5.1%)  4 (2.6%)  34 (21.9%)  9 (5.9%)  8 (5%)  1 (0.7%)  16 (10.3%)  32 (20.7%)  14 (9%)  13 (8.5%)  2 (1.3%)  3 (1.9%)  26 (16.8%) | <0.0001 |
